# Elisha G. Tucker, M.D.

**Published:** 1896-02

**Authors:** 


### ELISHA G. TUCKER, M.D.

*Resolved*, That the American Academy of Dental Science deeply regrets the loss by death of Elisha G. Tucker, M.D., an honorary member and one of its original founders.

That in a professional life, begun with the soundest known foundation, and carried through more than half a century with intelligent consistency, he has left a record of perseverance in principle worthy of remembrance.

That we shall greatly miss his venerable and genial companionship, and we would express our sympathetic condolence for the family of our departed member.

JACOB L. WILLIAMS,
FREDERICK N. SEABURY,
A. F. PRESTON,
*Committee.*

